# Development and Validation of the Yonsei Face Database (YFace DB)

**DOI:** 10.3389/fpsyg.2019.02626

**Published:** 2019-12-03

**Authors:** Kyong-Mee Chung, Soojin Kim, Woo Hyun Jung, Yeunjoo Kim

**Affiliations:** ^1^Department of Psychology, Yonsei University, Seoul, South Korea; ^2^Department of Psychology, Chungbuk National University, Cheongju, South Korea; ^3^Department of Psychology, University of California, Berkeley, Berkeley, CA, United States

**Keywords:** face database, picture stimuli, film clip, facial expression, validation

## Abstract

The purposes of this study were to develop the Yonsei Face Database (YFace DB), consisting of both static and dynamic face stimuli for six basic emotions (happiness, sadness, anger, surprise, fear, and disgust), and to test its validity. The database includes selected pictures (static stimuli) and film clips (dynamic stimuli) of 74 models (50% female) aged between 19 and 40. Thousand four hundred and eighty selected pictures and film clips were assessed for the accuracy, intensity, and naturalness during the validation procedure by 221 undergraduate students. The overall accuracy of the pictures was 76%. Film clips had a higher accuracy, of 83%; the highest accuracy was observed in happiness and the lowest in fear across all conditions (static with mouth open or closed, or dynamic). The accuracy was higher in film clips across all emotions but happiness and disgust, while the naturalness was higher in the pictures than in film clips except for sadness and anger. The intensity varied the most across conditions and emotions. Significant gender effects were found in perception accuracy for both the gender of models and raters. Male raters perceived surprise more accurately in static stimuli with mouth open and in dynamic stimuli while female raters perceived fear more accurately in all conditions. Moreover, sadness and anger expressed in static stimuli with mouth open and fear expressed in dynamic stimuli were perceived more accurately when models were male. Disgust expressed in static stimuli with mouth open and dynamic stimuli, and fear expressed in static stimuli with mouth closed were perceived more accurately when models were female. The YFace DB is the largest Asian face database by far and the first to include both static and dynamic facial expression stimuli, and the current study can provide researchers with a wealth of information about the validity of each stimulus through the validation procedure.

## Introduction

Facial expression plays an important role in the formation and maintenance of social relationships between individuals (McKone and Robbins, 2011). Researchers have investigated various aspects of face perception, including the mechanisms behind facial recognition and discrimination, information processing of faces, development of face perception, and the relationship between mental disorders and face recognition (Tsao and Livingstone, 2008; Calder et al., 2011). For example, it was found that infants prefer face-like stimuli to non-facial stimuli (Johnson et al., 2008) and that their face recognition skills become more sophisticated as they age (Passarotti et al., 2003; Golarai et al., 2007; Scherf et al., 2007). A plethora of fMRI studies have identified specific brain regions and networks, such as the fusiform face area, superior temporal sulcus, and occipital lobe, that are responsible for processing face perception (Ishai, 2008; Pitcher et al., 2011). In addition, people generally perform holistic than local processing of faces, meaning that parts of faces are integrated into one meaningful entity rather than separate parts perceived independently (Richler and Gauthier, 2014; Behrmann et al., 2015). Researchers have demonstrated that people develop a template for face perception and continuously modify it as they gain more experience (e.g., norm-based coding), rather than processing individual faces one by one (e.g., exemplar-based coding; Rhodes et al., 2005). Other studies have shown that the face perception differs across age, gender, and race, based on the information at hand (Zebrowitz et al., 2003, 2007; Hess et al., 2004; Becker et al., 2007). Moreover, some studies have shown significant correlations between deficits in face perception and various disorders, such as autism spectrum disorder (Dawson et al., 2005; Harms et al., 2010), and schizophrenia (Kohler et al., 2009).

In the studies of face perception and recognition, actual pictures of people were used as stimuli in computerized tasks to measure individual decision-making behaviors or brain activities via fMRI or ERP methods (Calder et al., 2011). These pictures were sometimes modified, depending on the purpose of the study, where some were composited or manipulated (Steyvers, 1999; Leopold et al., 2001) and others were altered to leave only the outlines of the faces (Wilson et al., 2002; Kim and Kim, 2003). Despite the variations in study goals, since most studies using facial stimuli require variability in their stimuli, it is extremely difficult for individual researchers to develop their own stimuli. Consequently, several researchers have developed face stimuli databases and have distributed them.

Table 1 summarizes the most widely used face databases. Each database typically consists of the six basic emotions defined by Ekman et al. (1972) which are happiness, sadness, anger, surprise, fear, and disgust, depicted by at least 20 models, although varying in their number and the types of stimuli. Upon permission, many researchers have conducted studies with selected stimuli from the databases. Pictures of Facial Affect (POFA) is one of the earliest face stimuli databases, developed by Ekman and Friesen (1976) and consisted of 110 black-and-white pictures expressing the six basic emotions. The most widely used database in the face perception studies is NimStim set (Tottenham et al., 2009), where many researchers have compared perceptual differences across diverse clinical populations (Norton et al., 2009; Hankin et al., 2010; Levens and Gotlib, 2010) or examined brain activities using an fMRI approach (Harris and Aguirre, 2010; Monk et al., 2010; Weng et al., 2011). Noted for its wide range in age of 179 models (ranging from the 20 s to the 60 s), the FACES database (Ebner et al., 2010) is mainly used in studies examining differences in face recognition ability across different age groups (Ebner and Johnson, 2009; Voelkle et al., 2012). The Chicago Face Database (Ma et al., 2015), which contains male and female, Black and White individuals in its initially published subset, was used in testing the effect of race in perceiving faces (Gwinn et al., 2015; Kleider-Offutt et al., 2017).

**TABLE 1:** Features of existing database.

**Country**	**Database**	**Author**	**Number of models**	**Number of stimuli**	**Types of stimuli**	**Other features**
Foreign	POFA (Pictures of Facial Affect)	Ekman and Friesen, 1976	14	110	Static stimuli: six basic emotions	Black and white Consisted only of Caucasian and African-American population
	
	JACFEE (Japanese and Caucasian Facial Expressions of Emotion)	Biehl et al., 1997	56	56	Static stimuli: six basic emotions + contempt	Included Japanese and Caucasian American
	
	JAFEE (The Japanese Female Facial Expression Database)	Lyons et al., 1998	10	213	Static stimuli: six basic emotions + neutral expressions	Consisted only of Japanese female models
	
	MSFDE (Montreal Set of Facial Displays of Emotion)	Beaupré et al., 2000	24	144	Static stimuli: happiness, anger, sadness, fear, disgust, shame + neutral expressions	Included French Canadian, Chinese, and sub-Saharan African
	
	TFEID (Taiwanese Facial Expression Image Database)	Chen and Yen, 2007	40	7200	Static stimuli: six basic emotions + contempt + neutral expressions Two different gaze angles (0, 45°) Two different camera angles (0, 45°) Two kinds of intensities (high, slight)	Consisted only of Taiwanese
	
	The CAS-PEAL-R1	Gao et al., 2007	1,040	30,863	Static stimuli: smile, frown, surprised, neutral, eye-closed, mouth-open expressions 15 illuminations four backgrounds six accessories	Black-and-white formats Consisted only of Chinese
	
	The NimStim set of Facial Expression	Tottenham et al., 2009	43	672	Static stimuli: six basic emotions + calmness + neutral expressions Except for surprised expressions, all other expressions were with mouth open or mouth closed Happy expression with three different versions (mouth closed, mouth open, high arousal mouth open)	Included various ethnic groups (Caucasian, African–American, Asian, Latin)
	
	FACES	Ebner et al., 2010	179	2,052	Static stimuli: five basic emotions except for surprise + neutral expressions	Included various age groups (youth, middle age, old age) Consisted only of Caucasian population
	
	RaFD (Radboud Faces Database)	Langner et al., 2010	67	6,030	Static stimuli: six basic emotions + contempt + neutral expressions Three different gaze directions (left, right, front side) Five different angles (180, 135, 90, 45, 0°)	Included Caucasian adults and children
	
	ADFES (Amsterdam Dynamic Facial Expression Set)	van der Schalk et al., 2011	22	648	Dynamic stimuli: six basic emotions + embarrassment, pride, contempt Three different angles (face-forward, turn-toward, turn-away)	Consisted of two ethnic groups: North European, Mediterranean
	
	The MPI Facial Expression	Kaulard et al., 2012	19	About 20,000	Dynamic stimuli: 56 expressions including fear, achievement, boredom	Raters had no professional experience
	
	The Chicago Face Database	Ma et al., 2015	158	158	Static stimuli: neutral expressions, fearful/afraid, angry, happy with closed mouth smile, happy with open mouth	Consisted of White and Black males/females Included additional information of the models from the raters’ perspective (attractiveness, babyfacedness, femininity, masculinity, Afrocentricity, trustworthiness, and unusual)
	
	WSEFEP (Warsaw Set of Emotional Facial Expression Pictures)	Olszanowski et al., 2015	30	210	Static stimuli: six basic emotions + neutral expressions	Consisted only of Polish population
	
	The EU-Emotion Stimulus set	O’Reilly et al., 2016	19	418	Dynamic stimuli: 20 emotions including six basic emotions + neutral expressions	Included various age groups Mainly Caucasian; included African–American and mixed ethnicities Included motor movements in social situations
	
	SAVE (Stills And Videos of facial Expressions)	Garrido et al., 2017	20	180	Static stimuli: laugh, frown, neutral expressions Dynamic stimuli: laugh, frown, neutral expressions Consisted of 5- and 10-s episodes	Undergraduate students participated in photographing
	
Korea	KFDB (Korean Face Database)	Hwang et al., 2004	1,000	52,000	Static stimuli: neutral expressions, happy, angry, surprised expressions Nine lights Seven different angles	Not validated
	
	PF07 (POSTECH Face Database)	Lee et al., 2008	200	64,000	Static stimuli: neutral expressions, happy, surprised, angry expressions Five different angles 16 lights	Not validated
	
	KUFEC (Korea University Facial Expression Collection)	Kim et al., 2011	49	5,880	Static stimuli: six basic emotions + two types of neutral expression Three different angles Five gazes	Validated based on Semantic Differential Method (Pleasure, arousal, dominance)
	
	KOFEE (the Korean Facial Expressions of Emotion)	Park et al., 2011	200	1,600	Static stimuli: six basic emotions + contempt + neutral expressions	Validated only for 176 stimuli
	
	KUFEC-II (Korea University Facial Expression Collection 2nd Edition)	Kim et al., 2017	57	399	Static stimuli: six basic emotions + neutral expressions Three different angles	
	
	Extended ChaeLee	Lee et al., 2013	50	283	Static stimuli: six basic emotions + neutral expressions	

Although limited, face databases containing Asian faces have also been developed. The Japanese and Caucasian Facial Expressions of Emotion (JACFEE) database (Biehl et al., 1997) has been used in various studies examining brain activity patterns across emotionality (Whalen et al., 2001), differences in facial expression recognition across cultures (Matsumoto et al., 2002), and differences in facial recognition ability of individuals with clinical disorders such as schizophrenia or social anxiety disorder (Hooker and Park, 2002; Stein et al., 2002). The female-only version, Japanese Female Facial Expression (JAFEE) Database (Lyons et al., 1998), is also available which includes pictures of Japanese female models expressing six basic emotions and neutral expressions. The CAS-PEAL-R1 database (Gao et al., 2007), a large-scale Chinese face database, consists of 30,863 pictures of 595 men and 445 women expressing six facial emotion expressions. The CAS-PEAL-R1 has been frequently used for face recognition studies in computer science (Zhang et al., 2009; Rivera et al., 2012). The Taiwanese Facial Expression Image Database (TFEID) (Chen and Yen, 2007) includes 7800 stimuli of 40 Taiwanese models expressing 6 basic emotions with two levels of intensity and neutral expressions. Montreal Set of Facial Displays of Emotion (MSFDE) (Beaupré et al., 2000) database is more diverse in terms of ethnic background of its 24 models, including Chinese, French Canadian, and sub-Saharan African. Still, only a small proportion from the total 144 stimuli are Asian face stimuli.

Recently, databases including film clips as well as pictures have been developed to capture and deliver the dynamics of emotions. For example, the Stills and Videos of facial Expressions (SAVE) database (Garrido et al., 2017) included pictures and film clips of models laughing and frowning, as well as presenting neutral expressions. The Amsterdam Dynamic Facial Expression Set (ADFES; van der Schalk et al., 2011) recorded film clips of the six basic emotions plus three subtle expressions, such as embarrassment, pride, and contempt, taken from three different angles. In addition, there are databases with various facial emotion expressions, such as the EU-Emotion Stimulus set, containing 20 different facial emotion expressions (O’Reilly et al., 2016), and Max Planck Institute Facial Expression (Kaulard et al., 2012), with 56 sets of facial emotion expressions.

In Korea, various face databases comprising only of static face stimuli have been developed and used in a number of studies. For example, the Korea University Facial Expression Collection (KUFEC; Kim et al., 2011) was used to examine the facial recognition ability of clinical populations (Jung et al., 2015; Jang et al., 2016; Kim and Kim, 2016) and the differences in perceiving facial expressions in a non-clinical adult group (Kim et al., 2013). Recently, KUFEC-II, a revised version of KUFEC, has been developed to overcome the limitations of KUFEC in its shooting and selection of stimuli by adopting the Facial Action Coding System (FACS; Ekman et al., 2002) Some Korean databases, such as Extended ChaeLee (Lee et al., 2013) and the Korean Facial Expressions of Emotion (KOFEE) database (Park et al., 2011) have been used in many neuroscience studies with emphasis on the facial perception skills of clinical populations (Kim et al., 2014; Lee et al., 2015; Oh et al., 2016; Park et al., 2016). Furthermore, other databases, such as the Korean Face Database (KFDB; Hwang et al., 2004) and the POSTECH Face Database (PF07; Lee et al., 2008), have been employed in developing algorithms for face recognition.

However, due to a few limitations of the existing databases, foreign or Korean, their employment in Korean research has faced some challenges. First, foreign databases are limited in the number and types of Asian facial emotion expressions contained within. For example, the CAS-PEAL-R1 does not include all six basic emotions in its database and the stimuli formats are in black-and-white, resulting in its limited usage. NimStim set includes only six Asian out of its 43 models, and MSFDE contains 8 Asian out of its 24 models. Although the JACFEE database includes 28 pictures of 14 Asian models, which is a half of its total number of stimuli, the pictures are only presented in the black-and-white format, again limiting its utilization. Plus, JAFEE only consists of female models, which may not be suitable to be used across genders (Thayer and Johnsen, 2000). Previous studies have shown that people are more likely to remember the faces of their own race than those of other races (O’toole et al., 1994; Walker and Tanaka, 2003) and to recognize emotions of the same race with higher accuracy (Kilbride and Yarczower, 1983; Markham and Wang, 1996). These findings suggest that the performance in face perception is influenced by the race of models in the stimuli, which suggests the need to use models for face stimuli of the same race as the rater in face perception research.

Second, the existing Korean face databases are not as diverse in the numbers and types of stimuli as foreign databases, restricting selections for studies, or not validated. Validation is an essential process in developing databases, which typically includes measuring the accuracy, intensity, and naturalness of the stimuli (Tottenham et al., 2009). Although the Extended ChaeLee is validated, it only includes frontal faces and has a relatively small number (50) of stimuli. Both KUFEC and KUFEC-II consist of a large number of stimuli taken from three different angles (45°, 0°, −45°) with five different viewpoints (front, up, down, left, and right) of two different neutral expressions and six basic emotions of 49 and 57 male and female Korean models. However, only 672 stimuli from KUFEC and 399 from KUFEC-II have been validated, and all are static stimuli, restricting its utilization for a range of studies. KOFEE is also validated for only 176 stimuli, which also limits selection for researchers. Moreover, although KFDB and PF07 include a large number of stimuli (52,000 for KFDB, 64,000 for PF07), they have not been validated as well.

Third, despite the dynamic nature of emotional expressivity and its perception in daily social interactions (Sato and Yoshikawa, 2004; Kamachi et al., 2013), most of the existing databases, especially the Korean databases, consist only of static stimuli, which raises the ecological validity issue (Rhodes et al., 2011; van der Schalk et al., 2011; Kościński, 2013). Indeed, recent studies revealed that study results are affected by the types of stimuli (static or dynamic). For example, some researchers found that facial expressions are perceived more accurately in dynamic than in static stimuli (Ambadar et al., 2005; Trautmann et al., 2009) and that the intensity and naturalness are rated higher in dynamic stimuli (Biele and Grabowska, 2006; Weyers et al., 2006; Cunningham and Wallraven, 2009). Furthermore, raters reported a higher arousal level when presented with dynamic rather than static stimuli (Sato and Yoshikawa, 2007a), and they imitated models’ facial expressions more accurately (Sato and Yoshikawa, 2007b; Sato et al., 2008). In addition, neuroimaging studies have noted that the range of brain neural networks activated when processing dynamic facial expressions is wider than that for static expressions. Thus, a database that subsumes both the static and dynamic stimuli is called for, and, internationally, several facial stimuli databases have been developed to include dynamic stimuli into their databases. Yet, such database that suits Asian population has not been developed.

The purpose of this study is to develop a new face database that encompasses both static and dynamic stimuli to complement for the limitations in the existing face databases for the Asian population.

## Materials and Methods

### Stimulus

#### Models

Models were recruited via advertisements posted on online meetup groups for models in both the general and college communities. A total of 74 models (37 males, 37 females, aged from 19 to 40 years) participated in the photo and film shoots. They were informed of the date, time, and location of shooting via text messages, email, or phone calls. On the day of the shooting, the models were provided with a brief description of the research study and procedure, and participated upon consenting.

#### Stimuli

For the Yonsei Face Database (YFace DB), static and dynamic stimuli displaying emotional and neutral faces were obtained. Static stimuli consisted of seven facial emotion expressions in total, including the neutral expression and six basic emotions: happiness, sadness, anger, surprise, fear, and disgust (Ekman et al., 1972). In addition, since the six basic emotions are expressed differently when the mouth is open or closed, each expression was taken in two conditions (mouth open and mouth closed). Neutral expressions with mouth open, mouth closed, and mouth and eyes closed were captured. Moreover, to be compatible with face recognition studies that featured various angles (Royer et al., 2016), the mouth-closed neutral expressions were taken at 45° and 90° angles from both left and right sides in addition to the frontal images and films.

As with the static stimuli, dynamic stimuli (films) featured the six basic emotion expressions (happiness, sadness, anger, surprise, fear, and disgust). The models were instructed to change their facial emotion expression from a neutral state to peak states of each emotion in 4–5 s. Neutral dynamic stimuli included head and gaze movements. The head movements were captured as models moved their heads from the front to the left side at a 90° angle then back to the front, and to the right side at a 90° angle and then back to the front. Each 90° angle movement took approximately 4–5 s, constituting 15–20 s for the whole movement. Gaze movements were filmed as models moved their eyes in vertical, horizontal, and diagonal directions and then back to the front. It took approximately 1–2 s to move the eyes to one direction and then revert to the front. In total, it took approximately 5–10 s for each film clip. Afterward, the head and gaze movements were edited for each direction to create the final sets of stimuli.

#### Procedures

**FIGURE 1:**
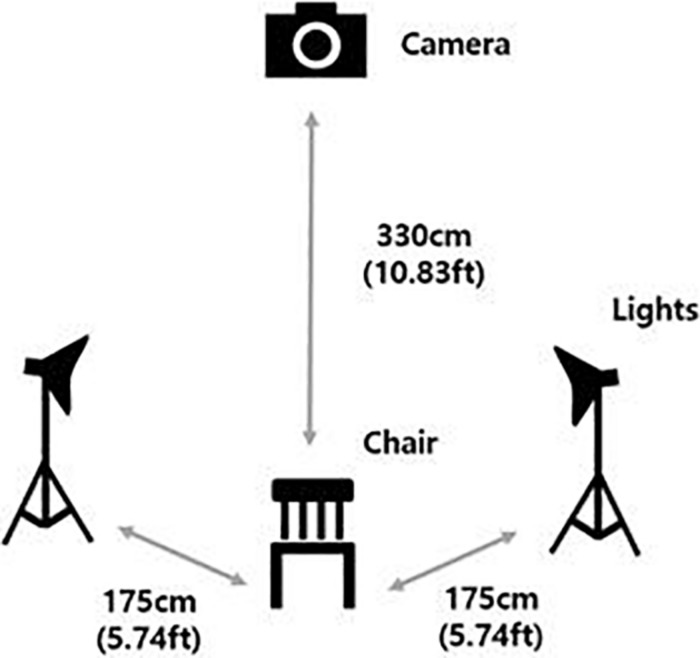
Setup of the photo shoot.

The photo and film shoots took place in a video production room at a university library located in Seoul. The models were instructed to sit in a chair in front of a white background screen. A Canon EOS 5D Mark II camera equipped with a Canon EF 70–200 mm lens was used, along with two PROSPOT DHL-1K standing lights. The distance between the chair and the camera was approximately 330 cm (10.83 ft), and the distance between the chair and the lights were approximately 175 cm (5.74 ft) each. Figure 1 shows the setup of the photo shoot.

First, the models were provided with the information about the purpose and details of the shooting. After they had consented to the procedure, models were asked to change into black T-shirts and remove any confounding features, such as beards, mustaches, necklaces, glasses, makeup, or bangs. Before the shooting, models were given a list of items to be pictured and filmed, with corresponding photo samples of each item to practice. Instructions were provided on the detailed facial muscle action units for the facial emotion expressions, as described in the Facial Action Coding Scheme of Ekman et al. (2002). Then photos per each emotion expression were taken in the order on the list while models were instructed to make facial emotion expression in front of the camera as naturally as possible. Models were also asked to make their expressions as intense as possible, because some researchers have reported that Koreans tend to express facial emotion with relatively weak intensity (Kim et al., 2011). A professional photographer directed the shooting with the help of a graduate student research assistant majoring in Psychology. They elicited the expressions from the models by describing situations most appropriate for each emotion. Static stimuli for each emotion were taken first, followed by neutral expressions with different head directions. Then, dynamic stimuli for each emotion were filmed, followed by neutral expressions with different head/gaze movements. The photographer took several pictures for each emotion until both the photographer and the research assistant agreed that the models made the most congruent facial expression to the requested emotion. Table 2 shows a categorized list of all stimuli included in the database.

**TABLE 2:** The structure of the YFace DB.

**Types**	**Emotions**	**Mouth shape**	**Direction of face**
Static stimuli	Happy	Mouth open	Front
	
		Mouth closed	Front
	
	Sad	Mouth open	Front
	
		Mouth closed	Front
	
	Angry	Mouth open	Front
	
		Mouth closed	Front
	
	Surprised	Mouth open	Front
	
		Mouth closed	Front
	
	Fearful	Mouth open	Front
	
		Mouth closed	Front
	
	Disgusted	Mouth open	Front
	
		Mouth closed	Front
	
		Mouth open	Front
	
	Neutral expressions	Mouth closed	Front
			90 degree angle to left side*
			45 degree angle to left side*
			90 degree angle to right side*
			45 degree angle to right side*
		Eyes closed + Mouth closed*	Front

Dynamic stimuli	Happy	-	Front
	
	Sad		Front
	
	Angry		Front
	
	Surprised		Front
	
	Fearful		Front
	
	Disgusted		Front
	
	Non-emotion* (Head movement)	-	Head to the left
			Head to the right
			Head to both sides
			Head up
			Head down
			Head both up then down
	
	Non-emotion* (Gaze)	-	Upper gaze
			Lower gaze
			Left gaze
			Right gaze
			Upper+Lower+Left+Right gaze
			Upper left gaze
			Upper right gaze
			Lower left gaze
			Lower right gaze
			Upper left+Upper right+Lower left+Lower right gaze

*^∗^Items marked above were not included in the validation process.*

#### The Stimuli Selection Procedure

**FIGURE 2:**
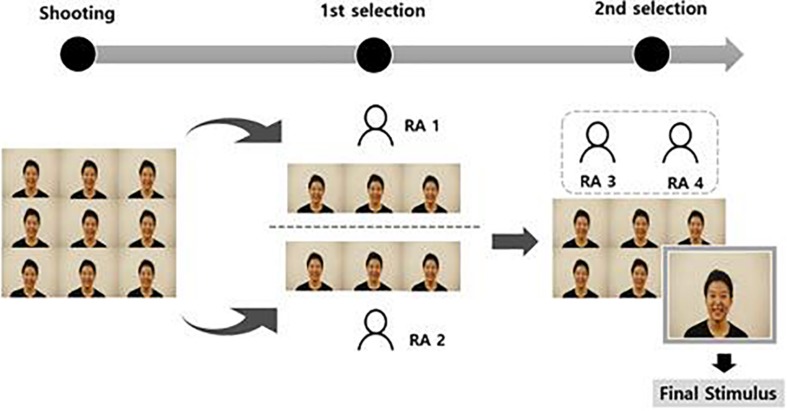
Process of selecting final stimuli for validation.

In order to select stimuli for the database, a two-step procedure was used (Figure 2). Two research assistants participated in the first process, and another two assistants participated in the second process. All research assistants were trained on the basic facial emotion expressions (Ekman et al., 2002) by the graduate student who participated in the shooting for an hour, and any questions that raised were addressed to minimize misunderstandings.

In the first part of the static stimuli selection procedure, two research assistants selected three static stimuli per facial emotion expression based on the following criteria. First, the stimuli should express each emotion appropriately and with high intensity (Ebner et al., 2010). Second, the stimuli should involve a head or body leaning at a minimal level. Third, the forehead should be well revealed in the stimuli. In the second part of the procedure, another two research assistants selected one stimulus from the three static stimuli per facial expression chosen from the first selection process, using the same criteria.

In the first part of the dynamic stimuli selection procedure, two research assistants selected one or two dynamic stimuli with head/gaze movement per facial expression according to following criteria. First, the stimuli should express each emotion appropriately with high intensity. Second, the stimuli should involve a head or body leaning toward or backwards at a minimal level (close to a 90° angle). Third, the stimuli should be presented with little body movement except for face turning. In the second part of the procedure, one set of dynamic stimuli for each facial expression with the head/gaze movement was selected according to the following criteria. First, dynamic stimuli should involve as little eye blinking as possible. Second, the stimuli should include the face turning at a 90° angle to both the left and right sides. Third, the head/gaze movements should comply with the speed timeframe (4–5 s to move the head to a 90° angle and 1–2 s to move gaze direction to one side and revert to the front). In the second part of the procedure, another two research assistants selected one stimulus out of the selected stimuli per facial expression, using the same criteria.

#### Editing the Stimuli

In the final stage of development, 1,480 stimuli (1,036 static stimuli and 444 dynamic stimuli) were selected from a total of 3,034 stimuli (1,406 static stimuli and 1,628 dynamic stimuli). The hue and size were edited for standardization using either Adobe Photoshop CS6 or Adobe Premiere CS. The static stimuli were adjusted to a resolution of 516 × 3444 and dynamic stimuli to 1920 × 1080. The neutral expressions of static stimuli with different head directions and dynamic stimuli with the head/gaze movements were separated according to the direction of head/gaze movement and saved separately.

### Validation Procedure of Stimuli

#### Participants

A total of 230 undergraduate students recruited from a university in Seoul (101 males, 120 females, aged from 18 to 28) participated in the database validation procedure. The participants were recruited via a recruitment website for research participants at the university in return for two course credits. Nine participants (five females) were excluded in the analysis due to technical errors during the data collection; hence, 221 participants were included in the final analysis. This study was approved by Yonsei University of Korea, Institutional Review Board (Approval No.: 7001988-201804-HR-140-09).

#### Materials

A sixth-generation i3 desktop with Windows 10 operating system and 22-inch monitor with a resolution of 1920 × 1080 was used in the experiment. The Psychopy 1.84.2 program was utilized to create computer-based experimental tasks and to record responses.

#### Stimuli

A total of 1480 ratable stimuli from the Yonsei Face DB were reviewed for evaluation (1036 static stimuli of six basic emotions and neutral expressions with mouth open or closed and 444 dynamic stimuli of six basic emotions). Static stimuli with neutral expressions taken from side angles and dynamic stimuli with head/gaze movements that were not suitable for assessing the accuracy, intensity, and naturalness were excluded in the validation process. The stimuli were resized to avoid errors in storing data online using Adobe Photoshop CS6 or Adobe Premiere CS. As a result, static stimuli with 657 × 438 pixels and dynamic stimuli with 640 × 360 pixels were used for the computerized tasks.

#### Validation Criteria

Items were evaluated for accuracy, intensity, and naturalness because such properties are the most widely measured criteria in validating a facial emotion expression database. Accuracy is the most foundational indicator for selecting stimuli and has been widely used by researchers in the development and validation of face databases, which measures what expression each facial emotion represents (Tottenham et al., 2009; Ebner et al., 2010). Intensity is the degree to which an individual is influenced by facial stimuli and is known to be one of the most salient aspects of emotion (Sonnemans and Frijda, 1994). Some studies have found that there is a correlation between the intensity and accuracy of an emotion expressed, where high intensity of facial expression is associated with higher accuracy in face perception. This suggests that intensity is also an important factor to be considered when selecting a stimulus (Palermo and Coltheart, 2004; Adolph and Georg, 2010; Hoffmann et al., 2010). The face databases that measure intensity include the Warsaw Set of Emotional Facial Expression Pictures (WSEFEP) database (Olszanowski et al., 2015) and the EU-Emotion Stimulus set (O’Reilly et al., 2016). Naturalness refers to the degree to which a facial stimulus truly reflects the emotion experienced at the moment (Livingstone et al., 2014). In particular, naturalness is highly correlated with the ecological validity (Carroll and Russell, 1997; Limbrecht et al., 2012). For example, people perceive natural smiles more positively than awkward smiles, indicating the dependence of facial expression perception on the naturalness of the expression (Miles and Johnston, 2007). One of the face databases that evaluated naturalness is the MPI Facial Expression Database (Kaulard et al., 2012).

To measure accuracy, the raters were asked to choose which label best represented the emotion displayed in each picture and in the film clips from choices of the six basic emotion expressions and neutral expressions (only the six basic emotion expressions for the clips). To measure the intensity and naturalness, a seven-point Likert-scale was used where 1 represented low intensity (or low naturalness/high awkwardness) and 7 represented high intensity (high naturalness).

#### The Procedure

**FIGURE 3:**
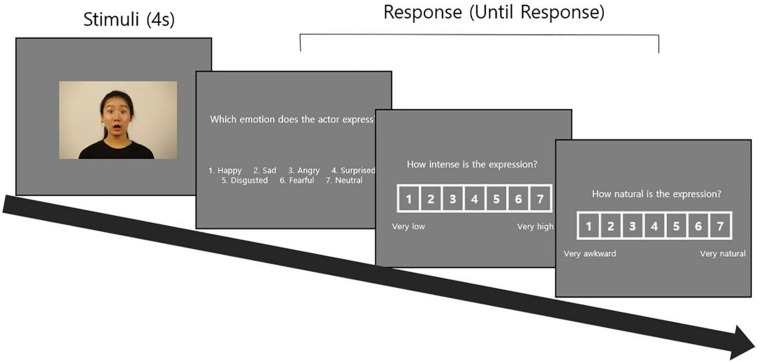
Validation procedure.

**FIGURE 4:**
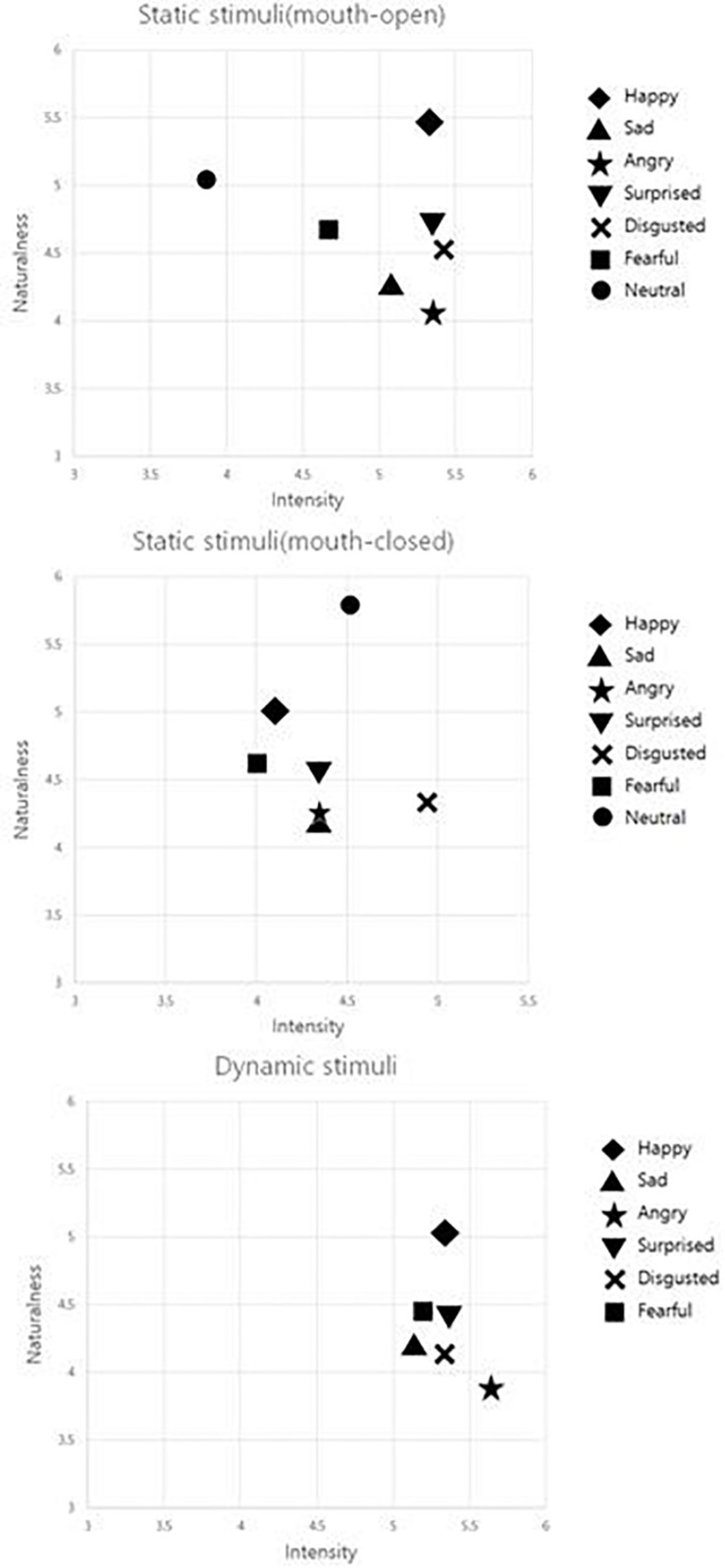
Relative positions of ratings in intensity and naturalness across emotions by stimuli type.

After being informed about the research study, the raters signed the consent form and participated in the procedure described below. To avoid the fatigue effect in raters, the number of stimuli evaluated per person was limited to 10% of the 1480 stimuli, so that the assessment could be completed within a 1 hr timeframe. The stimuli for the evaluation were therefore separated into ten sets consisting of 148 stimuli per set (a combination of 103 static stimuli and 45 dynamic stimuli or 104 static stimuli and 44 dynamic stimuli). The evaluation process was performed in two blocks, one for static stimuli and the other for dynamic stimuli. Raters were encouraged to take a break between the blocks if desired. To account for the order effect in presenting stimuli, the sequence between static and dynamic stimuli blocks was counterbalanced. Static stimuli were presented for 4 s each, followed by a question, “Which emotion does the actor express?” and choices for the six basic emotion and neutral expressions. Subsequently, questions regarding the level of intensity and naturalness were asked, and the raters responded to each question on the seven-point scale. For each question, the raters pressed a number from 1 to 7 on a keyboard to complete the response and then moved on to the next item (Figure 3). The same procedure was applied for the dynamic stimuli evaluation but only six choices of basic emotion expressions were given for assessing accuracy. Through this process, raters evaluated all 148 stimuli. Figure 4 shows relative positions of ratings in intensity and naturalness across emotions.

### Data Analysis

Descriptive statistics were conducted to calculate the means and standard deviations of accuracy, intensity, and naturalness for each stimulus.

A two-way ANOVA (3 × 6) was conducted with accuracy, intensity, and naturalness as dependent variables, and types of stimuli presented (three groups: static stimuli with mouth open or mouth closed, dynamic stimuli) and facial expressions (six emotions: happiness, sadness, anger, surprise, fear, and disgust) as independent variables. In addition, a Bonferroni *post hoc* analysis was performed for items with significant differences. In order to appraise the difference in the accuracy between the gender of models and the gender of raters, a two-way ANOVA (2 × 2) was conducted. In addition, a total of 14 independent *t*-tests were administered to examine the difference in the mean accuracy for six facial emotion expressions and neutral expression depending on the gender of models and raters. In this analysis, static stimuli with mouth open and mouth closed were combined.

## Results

The mean accuracy of the total facial stimuli included in the YFace DB was approximately 76% (*SD* = 42.60). The mean accuracy was 71% (*SD* = 45.40) for static stimuli with mouth open, 76% (*SD* = 42.78) for static stimuli with mouth closed, and 83% (*SD* = 37.90) for dynamic stimuli. The mean intensity was 5.02 (*SD* = 1.53) for static stimuli with mouth open, 4.38 (*SD* = 1.61) for static stimuli with mouth closed, and 5.35 (*SD* = 1.29) for dynamic stimuli. The mean naturalness was 4.67 (*SD* = 1.69) for static stimuli with mouth open, 4.68 (*SD* = 1.70) for static stimuli with mouth closed, and 4.34 (*SD* = 1.79) for dynamic stimuli. Table 3 shows the average scores for accuracy, intensity, and naturalness according to the type and facial emotion presented. In all types, happiness was rated with the highest accuracy (static stimuli with mouth open: 99.02% [9.84], static stimuli with mouth closed: 97.86% [14.47], dynamic stimuli: 99.08% [9.53]), indicating that the raters clearly distinguished happy facial expressions from other types of facial emotion expression. By contrast, fear was rated with the lowest accuracy of all types (static stimuli with mouth open: 26.93% [44.37], static stimuli with mouth closed: 30.47% [46.04], dynamic stimuli: 49.85% [50.02]), suggesting that the raters were more likely to confuse fearful facial expressions with other facial emotion expressions. Table 4 shows the percentage of each response choice per emotion (%).

**TABLE 3:** Average scores of stimuli in each emotion per stimulus type.

**Types**	**Static stimuli (mouth open)**			**Static stimuli (mouth closed)**			**Dynamic stimuli**		
			
**Emotions**	**Accuracy**	**Intensity**	**Naturalness**	**Accuracy**	**Intensity**	**Naturalness**	**Accuracy**	**Intensity**	**Naturalness**
	**(%)**	**(point)**	**(point)**	**(%)**	**(point)**	**(point)**	**(%)**	**(point)**	**(point)**
Happy	99.02(9.84)	5.34 (1.28)	5.46 (1.60)	97.86 (14.47)	4.10 (1.39)	5.01 (1.76)	99.08 (9.53)	5.35 (1.27)	5.01 (1.77)
Sad	76.04 (42.70)	5.09 (1.40)	4.25 (1.73)	82.53 (37.98)	4.35 (1.52)	4.17 (1.71)	90.40 (29.46)	5.15 (1.30)	4.18 (1.79)
Angry	86.71 (33.96)	5.37 (1.33)	4.04 (1.72)	87.23 (33.39)	4.35 (1.49)	4.26 (1.68)	94.74 (22.32)	5.65 (1.21)	3.87 (1.80)
Surprised	93.34 (24.94)	5.36 (1.31)	4.71 (1.64)	86.78 (33.88)	4.35 (1.49)	4.57 (1.61)	94.98 (21.83)	5.38 (1.28)	4.42 (1.78)
Disgusted	58.40 (49.30)	5.43 (1.30)	4.52 (1.64)	56.25 (49.62)	4.94 (1.44)	4.33 (1.66)	66.61 (47.18)	5.35 (1.35)	4.11 (1.77)
Fearful	26.93 (44.37)	4.68 (1.39)	4.66 (1.48)	30.47 (46.04)	4.01 (1.55)	4.62 (1.52)	49.85 (50.02)	5.20 (1.29)	4.44 (1.62)
Neutral	56.09 (49.64)	3.88 (1.93)	5.03 (1.61)	89.97 (30.05)	4.52 (2.09)	5.79 (1.35)			

*For intensity and naturalness, the score ranges from 1 to 7, with a higher score indicating higher intensity and naturalness.*

**TABLE 4:** Percentage of Each Response Choice per Emotion (%).

	**Happy**	**Sad**	**Angry**	**Surprised**	**Disgusted**	**Fearful**	**Neutral**
**Static Stimuli (mouth open)**							
Happy	99.0	1.5	1.2	1.2	0.1	0.4	0.5
Sad	0.5	76.2	1.3	0.5	20.3	7.2	3.4
Angry	0.1	3.2	86.7	1.5	13.2	10	6.7
Surprised	0.1	1.2	2.3	93.4	1.9	41.1	25.9
Disgusted	0.1	13.8	7.4	0.6	58.8	13.8	4.5
Fearful	0.2	4.0	1.0	2.8	5.6	26.9	2.8
Neutral	0.1	0.2	0.1	0.1	0.1	0.5	56.1
**Static Stimuli (mouth closed)**							
Happy	97.9	2.2	0.2	0.6	1.9	0.4	2.1
Sad	1.5	82.7	3.3	0.6	27.5	15	3.7
Angry	0.1	2.6	87.3	2.9	8.1	14.2	3.3
Surprised	0.2	0.5	0.7	86.8	1.2	22.7	0.2
Disgusted	0.1	8.7	6.8	1.2	56.7	14.5	0.3
Fearful	0	2.3	1.3	6.4	4.5	30.5	0.4
Neutral	0.2	1	0.4	1.5	0.2	2.6	90
**Dynamic Stimuli**							
Happy	99.1	1.8	0.1	0.4	0.7	0.5	
Sad	0.8	90.4	1.8	0.4	16.4	5.9	
Angry	0	1.9	94.7	0.9	8.3	5.2	
Surprised	0	0.5	0.7	95	1.8	25.9	
Disgusted	0	3.8	1.7	0.2	66.6	12.6	
Fearful	0.1	1.6	1	3.2	6.2	49.8	

In order to compare accuracy, intensity, and naturalness according to the types of stimuli and emotions, a two-way ANOVA was performed. Results showed a significant interaction effect between the types of stimuli and facial expressions across accuracy, *F*(10,29420) = 26.80, *p* < 0.001; intensity, *F*(10,29420) = 35.47, *p* < 0.001; and naturalness, *F*(10,29420) = 8.67, *p* < 0.001.

### Accuracy

The Bonferroni *post hoc* analysis (Table 5) showed that the accuracy of dynamic stimuli was significantly higher than that of the static stimuli, either with mouth open or closed, for all emotions except for the static stimuli of happiness with mouth closed and of surprise with mouth closed.

**TABLE 5:** Bonferroni *post hoc* analysis between types of stimuli.

			**Accuracy (%)**			**Intensity (point)**			**Naturalness (point)**		
					
**Emotions**	**Type a**	**Type b**	**Means difference of the two types**	***P***	**Cohen’s *d***	**Means difference of the two types**	***P***	**Cohen’s *d***	**Means difference of the two types**	***P***	**Cohen’s *d***
Happy	Static stimuli (mouth open)	Static stimuli (mouth closed)	1.16	0.01^∗∗^	0.09	1.24	< 0.001^*⁣**^	0.92	0.45	< 0.001^*⁣**^	0.27
	Static stimuli (mouth open)	Dynamic stimuli	–0.06	>0.05		–0.01	>0.05		0.45	< 0.001^*⁣**^	0.27
	Static stimuli (mouth closed)	Dynamic stimuli	–1.22	0.007^∗∗^	0.10	–1.25	< 0.001^*⁣**^	0.94	0	>0.05	
Sad	Static stimuli (mouth open)	Static stimuli (mouth closed)	–6.49	< 0.001^*⁣**^	0.16	9.74	< 0.001^*⁣**^	0,51	0.08	>0.05	
	Static stimuli (mouth open)	Dynamic stimuli	–14.36	< 0.001^*⁣**^	0.40	–0.06	>0.05		0.07	>0.05	
	Static stimuli (mouth closed)	Dynamic stimuli	–7.87	< 0.001^*⁣**^	0.23	–0.80	< 0.001^*⁣**^	0.57	–0.01	>0.05	
Angry	Static stimuli (mouth open)	Static stimuli (mouth closed)	–0.52	>0.05		1.02	< 0.001^*⁣**^	0.72	–0.22	< 0.001^*⁣**^	0.13
	Static stimuli (mouth open)	Dynamic stimuli	–8.03	< 0.001^*⁣**^	0.28	–0.28	< 0.001^*⁣**^	0.22	0.17	0.018^∗^	0.10
	Static stimuli (mouth closed)	Dynamic stimuli	–7.51	< 0.001^*⁣**^	0.26	–1.30	< 0.001^*⁣**^	0.96	0.39	< 0.001^*⁣**^	0.22
Surprised	Static stimuli (mouth open)	Static stimuli (mouth closed)	6.56	< 0.001^*⁣**^	0.22	1.01	< 0.001^*⁣**^	0.72	0.14	0.049^∗^	0.09
	Static stimuli (mouth open)	Dynamic stimuli	–1.64	>0.05		–0.02	>0.05		0.29	< 0.001^*⁣**^	0.17
	Static stimuli (mouth closed)	Dynamic stimuli	–8.2	< 0.001^*⁣**^	0.29	–1.03	< 0.001^*⁣**^	0.74	0.15	0.04^∗^	0.09
Disgusted	Static stimuli (mouth open)	Static stimuli (mouth closed)	2.15	>0.05		0.49	< 0.001^*⁣**^	0.36	0.19	0.003^∗∗^	0.12
	Static stimuli (mouth open)	Dynamic stimuli	–8.21	< 0.001^*⁣**^	0.17	0.08	>0.05		0.41	< 0.001^*⁣**^	0.24
	Static stimuli (mouth closed)	Dynamic stimuli	–10.36	< 0.001^*⁣**^	0.21	–0.41	< 0.001^*⁣**^	0.29	0.22	< 0.001^*⁣**^	0.13
Fearful	Static stimuli (mouth open)	Static stimuli (mouth closed)	–3.54	>0.05		0.67	< 0.001^*⁣**^	0.46	0.04	>0.05	
	Static stimuli (mouth open)	Dynamic stimuli	–22.92	< 0.001^*⁣**^	0.48	–0.52	< 0.001^*⁣**^	0.39	0.22	< 0.001^*⁣**^	0.14
	Static stimuli (mouth closed)	Dynamic stimuli	–19.38	< 0.001^*⁣**^	0.40	–1.19	< 0.001^*⁣**^	0.83	0.18	0.003^∗∗^	0.11
Neutral	Static stimuli (mouth open)	Static stimuli (mouth closed)	–33.88	< 0.001^*⁣**^	0.83	–0.64	< 0.001^*⁣**^	0.32	–0.76	< 0.001^*⁣**^	0.51

*^∗^p <0.05, ^∗∗^p <0.01, ^∗∗∗^p <0.001. The neutral expressions were not included in the ANOVA analysis.*

Of the two types of static stimuli (mouth open and mouth closed), differences in mean levels of accuracy were reported across different emotions. For happiness and surprise facial expressions, static stimuli with mouth open had a higher accuracy than the stimuli with mouth closed. In contrast, anger, disgust, and fear did not show significant differences in accuracy between mouth-open and mouth-closed conditions. Sadness and neutral expressions with mouth closed were more accurate than with mouth open.

### Intensity

The Bonferroni *post hoc* analysis (Table 5) showed that the intensity of anger was significantly higher in the dynamic stimuli than in the static stimuli with mouth closed and mouth open. The intensity for happiness, sadness, surprise, and disgust were higher in the dynamic stimuli than in the static stimuli with mouth closed, but there was no significant difference in the intensity in the static stimuli with mouth open. The neutral facial expression with mouth closed had a significantly higher intensity than that with mouth open.

### Naturalness

The Bonferroni *post hoc* analysis (Table 5) showed that the naturalness of the static stimuli with mouth open for all six emotions except sadness was significantly higher than that of dynamic stimuli. For anger, surprise, disgust, and fear, the static stimuli with mouth closed were rated as more natural than the dynamic stimuli were. There was no significant difference in the naturalness of the sadness expression between the dynamic stimuli and static stimuli. For anger and neutral expressions, static stimuli with mouth closed were rated as more natural than with mouth open.

### Differences in Accuracy Depending on Model and Rater Gender

The results showed that a significant difference in accuracy between the gender of raters was found for some emotion expressions. First, male raters perceived surprised expressions more accurately than did female raters, *t*(1634) = 2.18, *p* = 0.03, Cohen’s *d* = 0.11, while female raters perceived fearful expressions more accurately than did male raters in static stimuli with mouth open, *t*(1628) = −3.79, *p* < 0.001, Cohen’s *d* = 0.19. In static stimuli with mouth closed, the female raters perceived only the fearful expression more accurately than male raters did, *t*(1629) = −4.19, *p* < 0.001, Cohen’s *d* = 0.21. Meanwhile, the surprised expression in dynamic stimuli was perceived more accurately by the male raters, *t*(1633) = 3.30, *p* < 0.001, Cohen’s *d* = 0.16, whereas the fearful expression was perceived more accurately by the female raters than the male raters, *t*(1633) = −3.83, *p* < 0.001, Cohen’s *d* = 0.19.

Likewise, some emotion expressions were perceived more or less accurately depending on the gender of models. The accuracy for sadness, *t*(1634) = 4.84, *p* < 0.001, Cohen’s *d* = 0.24, and anger, *t*(1631) = 2.00, *p* = 0.046, Cohen’s *d* = 0.10, was higher in static stimuli with mouth open when the models were male. For the disgusted expression, accuracy was higher when the models were female, *t*(1630) = −5.69, *p* < 0.001, Cohen’s *d* = 0.28. In the static stimuli with mouth closed, fearful expressions were perceived more accurately when the models were female, *t*(1629) = −3.05, *p* = 0.002, Cohen’s *d* = 0.15. In the dynamic stimuli, the fearful expression was perceived more accurately when the models were male, *t*(1626) = 3.68, *p* < 0.001, Cohen’s *d* = 0.18. For the disgusted expression, accuracy was higher when the models were female, *t*(1633) = −5.59, *p* < 0.001, Cohen’s *d* = 0.28.

However, there was no significant interaction effect between the gender of the models and the raters across all emotions: static stimuli with mouth open *F*(1,11443) = 1.55, *p* > 0.05; static stimuli with mouth closed *F*(1,11444) = 0.44, *p* > 0.05; dynamic stimuli *F*(1,9808) = 0.08, *p* > 0.05.

## Discussion

The purpose of this study was to develop a Korean face database comprising both static and dynamic facial emotion expressions to complement for the limitations in the existing face databases. Six basic emotions and neutral facial expressions were captured in 1480 sets of static and dynamic stimuli, taken from 74 models and rated by 221 participants for the accuracy, intensity, and naturalness of the expressions. Results showed a high level of accuracy, an average of 76%, which is similar to that of other existing databases, and the mean intensity and naturalness of the stimuli were reasonable, at 4.89 and 4.57 out of 7, respectively. This study suggests a wide applicability of the database in research because its diverse array of stimuli, including not only dynamic motion clips but also static stimuli with mouth open and mouth closed, were successfully validated. The implications of the study are as follows.

First, it appears that the accuracy of perceiving facial expression varies across emotions. For example, while the highest accuracy was reported in perceiving happy facial expressions, the lowest accuracy was observed in perceiving fearful facial expressions, regardless of the stimuli type presented. These results are similar to previous studies that used the existing domestic face databases (Extended ChaeLee, happy 95.5%, disgusted 69.1%, and fearful 49%; KUFEC-II, happy 97.11%, disgusted 63.46%, and fearful 49.76%). Although the validated foreign face databases reported a higher accuracy in perceiving disgust and fear than the domestic face databases did, the accuracy of these two facial expressions are still relatively lower than those of other facial emotion expressions (JACFEE: happy 98.13%, disgusted 76.15%, and fearful 66.73%; NimStim set: happy 92%, disgusted 80%, and fearful 60%; Radboud Faces Database (RaFD): happy 98%, disgusted 80%, and fearful 81%). Thus, it can be inferred that the low accuracy of disgust and fear may not be a unique feature of the current face database but a common issue arising from the differences in face recognition in general. In particular, analyses of the incorrect responses of each emotion have shown that raters tend to confuse disgust with sadness and fear with surprise. In regard to this confusion, Biehl et al. (1997) offered several explanations. First, the morphological similarity between facial emotion expressions could influence the ability to distinguish fear from surprise expressions. For example, fearful and surprised expressions share some features, such as raised eyebrows, dilated pupils, and opened mouth (Ekman et al., 2002). Second, individual differences in the frequency of exposure to each facial emotion expression in everyday life could affect the results. For example, when interacting with other people in social settings, sad expressions are more frequently observed than disgusted expressions, and surprised expressions are more commonly encountered than fearful expressions. It could be assumed that people are more likely to confuse ambiguous facial emotion expressions with expressions that they are more familiar with given limited information available at hand. Third, since disgusted and fearful facial expressions use more facial muscles than other types of facial expressions do and require expressional elements that are more complicated, models may not had been able to express such emotions accurately, or raters may have had difficulty interpreting them correctly. In addition, cultural differences in face recognition may have an important impact on the results of this study. Previous studies have examined the cultural differences in perceiving facial expressions and found that Asians showed significantly lower recognition accuracy rates for fearful and disgusted expressions than did Western people when the same facial emotion expressions were presented (Ekman et al., 1987; Huang et al., 2001; Beaupré and Hess, 2005). Relatedly, given the relative difficulty that Asians have with facial recognition for fear and disgust, the validation of the current face database may have been undermined.

Second, significant differences in accuracy, intensity, and naturalness across emotions and types of stimuli suggest the need for careful selection of facial stimuli depending on the purpose of the study. For example, the accuracy of dynamic stimuli was higher than that of static stimuli for most facial emotion expressions. This is consistent with previous studies showing that dynamic stimuli are better tools for perceiving facial expressions accurately than static stimuli are (Ambadar et al., 2005; Trautmann et al., 2009). Unlike static stimuli, in which only one moment of an expression is presented, dynamic stimuli provide more information about how facial expressions gradually change over time (Krumhuber et al., 2013). In addition, even within the static stimuli, there was a difference in accuracy, depending on the shape of mouth, in some facial emotion expressions. In the case of happiness and surpised expressions, the accuracy was higher with the mouth open than with the mouth closed. For anger, disgust, and fearful expressions, however, no difference was found in the accuracy between open mouth and closed mouth stimuli. These results are different from those found in the NimStim study (Tottenham et al., 2009), which showed that the higher accuracy in sad expressions was observed with closed mouth stimuli and that the higher accuracy in happy, angry, and fearful expressions were found with open mouth stimuli. Happy and surprised facial expressions, which were rated more accurately with open mouth stimuli in the current study, were categorized as positive or neutral expressions, in contrast to angry, disgusted, and fearful facial expressions (Kim et al., 2011). This can be interpreted as for Asians, the effect of mouth shapes on negative expressions is weaker than for positive or neutral expressions, since the intensity of static stimuli with mouth open is higher overall than those with mouth closed. In previous studies, angry facial expressions with high intensity were perceived more accurately by Western people than by Asians, but the results showed that there was no difference in the accuracy between these two groups when an angry facial expression was presented with low intensity (Matsumoto, 1992; Biehl et al., 1997; Beaupré and Hess, 2005; Bourgeois et al., 2005). Further research should be conducted to test for this assumption by examining differences in facial emotion expression recognition between Asian and Western cultures.

By contrast, there was no significant difference between static stimuli and dynamic stimuli in terms of intensity. However, it is notable that both accuracy and intensity were significantly higher for angry and fearful expressions in dynamic stimuli than in the static stimuli. Moreover, the degree of additional information provided by motion clips for recognizing emotion expression varies by the type of emotion (Nusseck et al., 2008; Cunningham and Wallraven, 2009). The current study suggests that dynamic stimuli provide relatively more information for recognizing angry and fearful expressions than other types of facial emotion expression. Particularly, fearful facial expressions, for example, whereas the accuracy with static stimuli was less than 30%, the accuracy with dynamic stimuli was approximately 50%. Although accuracy at 50% is not considered high, considering the remarkably low accuracy for recognizing fearful facial expressions generally, utilizing dynamic stimuli rather than the static stimuli for fearful expressions is highly recommended in order to obtain reliable results in accordance to the research purposes.

As for naturalness, happy, surprised, disgusted, and fearful facial expressions were rated as more natural in static stimuli with mouth open than were dynamic stimuli. Databases containing dynamic facial stimuli tend to adopt one of two shooting methods (Scherer and Bänziger, 2010; Krumhuber et al., 2017). The first method is to provide specific instructions for creating facial emotion expressions. This method has the advantage of reducing variability in an actor’s facial emotion expression, making facial recognition easier, and enhancing the consistency across stimuli, but it has the disadvantage that it results in reduced naturalness in the stimuli. The second method involves shooting facial expressions that occur in a more natural situation, without giving any additional instructions. The advantage of this method is that the stimuli produced are perceived as more natural as they resemble with the facial emotion expressions observed in daily life. However, the consistency among facial expressions is compromised due to the difficulty in controlling them. This study adopted the first method, specifying instructions for each emotion when models were asked to make facial emotion expressions that changed from neutral to peak states of an emotion and controlling for any variability other than facial expression. Thus, the dynamic stimuli comprised footage of unnatural emotionality that is not easily observed in everyday life. It may be necessary to find a better way of incorporating the naturalness of facial expressions that reflect everyday interaction when developing new face databases in the future.

In terms of neutral expression, all evaluative criteria, such as accuracy, intensity, and naturalness, were met with higher scores for static stimuli with mouth closed than with mouth open. This may be because the expression that is generally perceived as a neutral expression is a form with mouth closed. In addition, by analyzing the incorrect responses, researchers found that the neutral expression with mouth open was confused with a surprised expression with weak intensity. Therefore, it is more appropriate to use static stimuli with mouth closed when the neutral expression is used for the stimuli in experiments.

Finally, although there was no significant interaction between the gender of the models and the gender of the raters on the accuracy of the total facial emotion expression in each stimulus, there was a significant difference in accuracy for some facial emotion expressions according to the gender of models or the raters. The significant gender difference for the models means that certain expressions were more accurately expressed by one gender of model. Considering these results, if stimuli of only one gender are used in an experiment, it may be appropriate to use the stimuli of the gender reported with higher accuracy according to each expression. Moreover, there was a difference in recognition accuracy according to the gender of the raters. Regardless of the type, the fearful expression was more accurately perceived by female raters, while the surprised expression was perceived more accurately by males. These results may be due to the tendency for females to be more aware of subtle and complex emotions than males are (Hoffmann et al., 2010). As we have seen above, fear is a more complex emotion to express than other types of facial emotion; thus, females may perceive fearful facial expressions better than males do. In a study comparing facial expressions between genders, it was shown that females perceived fearful facial expressions more accurately than males did (Mandal and Palchoudhury, 1985; Nowicki and Hartigan, 1988). On the other hand, the finding that females were less accurate in recognizing surprised expressions than males were suggests that females were biased toward fearful expressions in perceiving the emotion of surprise. However, there is limited research on the mechanism behind differences in perceiving surprised or fearful expressions between genders. Some neuropsychological studies have observed that females show a higher activation in the left amygdala than males when seeing fearful expressions (Thomas et al., 2001; Williams et al., 2006; Kempton et al., 2009). In this regard, it can be assumed that females are more sensitive to receiving emotions associated with fear. On the basis of the results of this study, further research may shed light on the underlying mechanisms behind the gender differences observed in perceiving different types of emotion expressions.

The limitations of this study are as follows. First, because both models and raters in this study were either in their 20 s or 30 s, the generalizability of the study results may be limited. Previous studies have noted an own-age bias, where people perform better in identity recognition (Wright and Stroud, 2002; Lamont et al., 2005; Anastasi and Rhodes, 2006) as well as emotion recognition in faces (Ebner and Johnson, 2010; Ebner et al., 2011) of the same age group than those of other age groups, suggesting the need to match face stimuli to the age group of participants in experiments. For this reason, face databases including models from various age groups, ranging from children to the elderly, have been developed recently (Ebner et al., 2010; Egger et al., 2011; Dalrymple et al., 2013). In particular, the FACES (Ebner et al., 2010) database has shown in its validation study that the stimuli of each age group were perceived differently across all age groups. However, in Korea, since there are only few databases that include a variety of age groups, it will be meaningful to form a group of models and raters to represent a wide range of age groups when developing a new face database in the future.

Second, in this study, the forced choice method was used to measure the accuracy of the stimulus in the validating procedure. This method seems to increase rates of incorrect responses for the facial recognition of the database overall by allowing the participant to select a response even when the stimulus is not clearly recognized. In some of the existing databases, a “none of above” or “other” option was added for the raters to choose from if the presented facial expression was not clearly applicable to any options listed (Tottenham et al., 2009; Langner et al., 2010). In a recently developed database, several facial expressions were presented in a continuous scale so that the participants were freed from the pressure of selecting a single categorical response when perceiving facial expressions (Olszanowski et al., 2015). Alternatively, a free-labeling method (Widen and Russell, 2010), which increases raters’ self-reflection to emotional stimuli, could be considered as a possible approach for measuring facial emotion expressions.

Using suggested methods to evaluate stimuli for the future development and validation of new databases may provide more detailed and accurate information for each stimulus. Third, in this study the confounding variables of models, such as the bangs, beard, dyed hair, and makeup, were removed to improve the consistency of the stimulus. However, in everyday life, it is common and natural to encounter such variables. This database may be useful for experiments that require consistent stimuli, but it may be less useful for experiments that prefer naturalistic stimuli. In order to increase the ecological validity of the stimulus, an alternative approach may be to shoot photos and films without removing the confounding variables. Finally, The YFace DB includes only Korean models, which could raise concerns regarding its use with other Asian ethnic groups or generalization of its results across cultures. Considering the possibility of subtle variation in codifying and expressing emotions in different cultures (Marsh et al., 2003; Elfenbein et al., 2007), further investigation of face recognition among other Asian ethnic groups using YFace DB should be followed.

The Yonsei Face DB is the first Asian face database to include static and dynamic stimuli for facial emotion expressions, providing abundant information for validation to help researchers select appropriate stimuli. It is expected that this database will be utilized in various fields related to face research and contribute to the development of related fields.

## Data Availability Statement

We confirmed that our data/materials are available upon request on our website (http://yonseipsye.dothome.co.kr/?p=758 or http://yonsei.au1.qualtrics.com/jfe/form/SV_a5WpsFtN4oaYPAh).

## Ethics Statement

The studies involving human participants were reviewed and approved by Yonsei University Institutional Review Board (Approval No.: 7001988-201804-HR-140-09). The patients/participants provided their written informed consent to participate in this study. Written informed consent was obtained from the individual(s) for the publication of any potentially identifiable images or data included in this article.

## Author Contributions

K-MC and WJ developed the theoretical framework and designed the experiments. K-MC directed the project. SK performed the experiments and analyzed the data. YK aided in interpreting the results and worked on the manuscript. K-MC, SK, and YK wrote the article.

## Conflict of Interest

The authors declare that the research was conducted in the absence of any commercial or financial relationships that could be construed as a potential conflict of interest.
